# Integrated transcriptomic and proteomic analyses reveal potential mechanisms linking thermal stress and depressed disease resistance in the turbot *Scophthalmus maximus*

**DOI:** 10.1038/s41598-018-20065-1

**Published:** 2018-01-30

**Authors:** Xin Yue, Pin Huan, Yonghua Hu, Baozhong Liu

**Affiliations:** 1 Key Laboratory of Experimental Marine Biology, Institute of Oceanology, Chinese Academy of Sciences, 7 Nanhai Road, Qingdao, 266071 China; 2 Laboratory for Marine Biology and Biotechnology, Qingdao National Laboratory for Marine Science and Technology, 1 Wenhai Road, Qingdao, 266000 China

## Abstract

A worldwide increase in the reports of diseases affecting marine organisms has paralleled the climate warming over the past few decades. In this study, we applied omics to explore the mechanisms underlying thermo-linked epizootics, by comparing both the transcriptome- and proteome-wide response of turbots to a mimic pathogen (poly I:C) between high temperature and low temperature using a time-course approach. Our results showed that myeloperoxidase (MPO) and insulin were differentially expressed transcripts shared by all five time-points post poly I:C-injection between high and low temperature and also had a consistent expression trend as differentially expressed proteins at 24 h post injection. Combined with other data, it was suggested that the elevated temperature enhanced neutrophil-mediated immunity and the resultant MPO-mediated oxidative stress, which lasted for at least 5 days. The contents of malondialdehyde and protein carbonyls, markers of oxidative damage for lipids and proteins, respectively, were compared between different temperature groups, and the results further implied the emergence of oxidative damage under high temperature. It was also suggested that metabolism disorder likely occur considering the sustained expression changes of insulin. Hence, prolonged MPO-mediated oxidative stress and metabolic disorder might be involved in the thermo-linked epizootic.

## Introduction

Global warming may be the most important environmental problem the world faces^1–3^. During the last 20 years, seawater temperatures throughout much of the globe have been elevated at unprecedented rates, accompanied by increased frequencies and magnitudes of extreme thermal events^4,5^. Notably, a worldwide increase in the reports of diseases affecting marine organisms has paralleled the seawater warming over the past few decades^6,7^. It has been demonstrated that temperature exerts a considerable influence on host disease resistance^8^. Thus, thermal stress induced by the current trend toward a warming climate that marine organisms are undergoing or will undergo may make the marine organisms more susceptible to disease, thereby increasing the frequency of opportunistic diseases.

Fish are important poikilothermic organisms in marine ecosystems, whose physiological function is directly affected by the ambient water temperature^9^. Elevated water temperature could influence the immune response of fish^10–12^, and has challenged fish by temperature-linked epizootics, where mortality likely resulted from the effects of thermal stress and an unidentified opportunistic pathogen^13–18^. The turbot *Scophthalmus maximus* is a cold-water flatfish that is naturally distributed around the European coast and also exists in the Southeast Pacific Ocean (Chile) and in China, where it has been introduced for farming. It inhabits shallow waters mainly during larval and juvenile stages and moves to deep waters upon reaching adulthood^19^. The potential influence of water temperature fluctuation, especially to the health of larval and juvenile turbots, should be considered. For the wild turbot, although virus-induced disease outbreak has been observed^20^, there are few records about the relationship between thermal stress and disease appearance. However, this relationship has been observed in the farmed turbot^21–23^, and it has been reported that the disease-induced mortality increased rapidly when the water temperature was beyond 20 °C^24^. It is therefore likely that pathogens affecting wild turbot populations will experience similar climate-driven changes.

This study focused on exploring the mechanisms underlying the correlation between thermal stress and depressed disease resistance in the turbot. Two ecologically relevant water temperatures were chosen in this study. Based on the monthly temperature record for the main natural habitats of the turbot juvenile, the upper limit was close to 21 °C (20.8 ± 0.4 °C)^25–27^, so 21 °C was chosen to model the extreme high temperature condition mediated by the climate change. Additionally, 15 °C, which is within the optimal temperature for the turbot juvenile^28^, was chosen as a nonstressful temperature. To be distinguished in the following paragraphs, 21 °C was designated as “high” temperature, while 15 °C was designated as “low” temperature. Besides the temperature setting, modeling a pathogen invasion condition is also needed. It has been indicated that climate warming may also affect the pathogen’s development and survival rates, except for the host susceptibility^8^. Here, we focused on the response of the host fish itself, not the response to the warming-induced changes in the quantity and virulence of a specific pathogen. Thus, polyinosinic:polycytidylic acid (poly I:C), which is a synthetic double-stranded RNA and has been broadly used experimentally to model viral infection in fish^29,30^, was applied as a pathogen mimic in this study. The spleen is regarded as a major immune organ in fish. As a major lymphoid organ, it plays an important role in protecting against pathogens during disease processes^31^ and thereby is choose as the target organ in this study to detect the response changes to the mimic pathogen poly I:C under different temperature.

The achievement of genome-wide gene expression quantification has allowed investigators unprecedented ability to understand the global molecular regulation mechanism when facing different environments^32^. To fully understand the synergies between thermal stress and disease on the fitness of the turbot at both the transcriptional and translational levels, we performed comprehensive transcriptomic analyses by RNA-seq and proteomic analyses by iTRAQ (isobaric tags for relative and absolute quantitation). Additionally, a time-course design (i.e., five time-points post poly I:C-injection) that could uncover the temporal expression patterns was adopted, allowing a better description of the entirety of the response and teasing apart general response genes from major regulatory genes^33^.

To our knowledge, this is the first study using omics to reveal the synergies between thermal stress and disease in the fish, by comparing both the transcriptome- and proteome-wide response of the turbot to a mimic pathogen (poly I:C) between high temperature (21 °C) and low temperature (15 °C).

## Results

### General information of transcriptome

**Figure 1:**
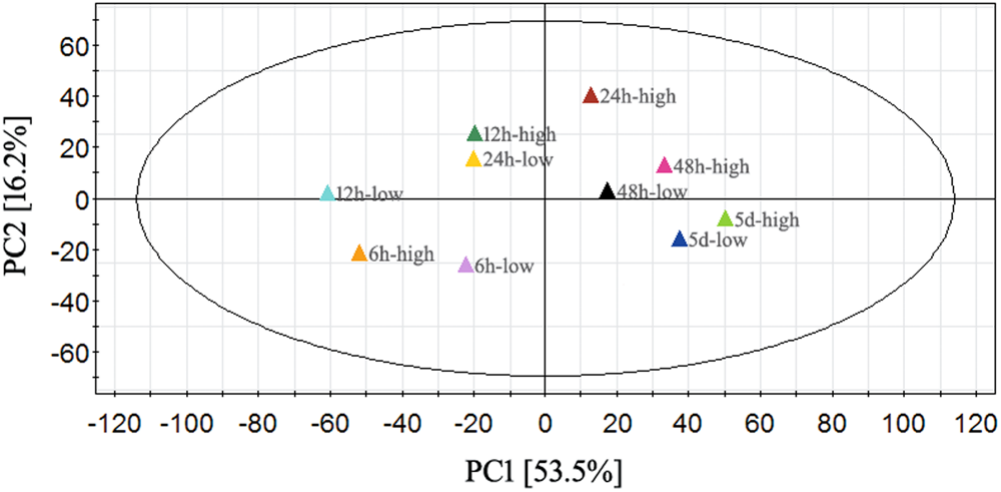
Principal component analysis of global mRNA expression in the turbot at different time points post poly I:C-injection under high or low water temperature.

To the turbot spleen reference *de novo* transcriptome, paired-end reads were generated, among which the left-end reads generated were pooled into one large left.fastq file named Spleen_l, while the right-end reads were pooled into one large right.fastq file named Spleen_2. The clean data of Spleen_l and Spleen_2 are available via NCBI with the accession number SRR4853423. In total, 136,440,468 clean reads filtered from 149,760,036 raw reads were generated through Illumina sequencing and were assembled into 134,481 unigenes with a mean length of 768 bp. These unigenes were annotated, among which 53,529 genes were annotated in NR, and 61,544, 39,524, 47,524, 32,639, and 17,240 genes were annotated in NT, KO, Swiss-Prot, GO and COG, respectively. The total clean reads of each of the ten treatment groups and two control groups were mapped back to the reference *de novo* transcriptome. The related information and clean data SRA accession numbers (NCBI) of each group are listed in Table 1. The global mRNA expression profiles of the ten treatment groups were compared by PCA (Fig. 1). The PCA results showed that there was a longer distance between the high and low temperature groups at 6 hpi, 12 hpi or 24 hpi than that at 48 hpi and 5 dpi. Thus, the transcriptional change between the high and low temperature groups was more drastic before 24 hpi than that after 24 hpi. In addition, according to the value of PC1 which explained more variability (53.5%), it was showed that 6 h-high & 12 h-low, 12 h-high & 24 h-low, 24 h-high & 48 h-low, 48 h-high & 5 d-low were closer to each other than the others, especially 12 h-high & 24 h-low, suggesting that elevated temperature hastened the transcriptional response.

**Table 1:** Information and accession number of RNA-seq.

Sample ID	Total clean reads	Total mapping ratio	SRA accession No.
0 h-high	12,616,966	93.35%	SRR6075019
6 h-high	12,046,282	90.18%	SRR4853431
12 h-high	12,098,564	91.58%	SRR4853443
24 h-high	12,320,035	90.89%	SRR4853440
48 h-high	11,732,782	89.18%	SRR4853446
5 d-high	11,938,080	90.52%	SRR4853429
0 h-low	12,456,513	92.16%	SRR6075022
6 h-low	11,908,034	90.93%	SRR4852900
12 h-low	12,519,896	91.27%	SRR4853434
24 h-low	11,679,987	91.14%	SRR4853437
48 h-low	11,831,024	90.95%	SRR4853426
5 d-low	12,170,353	92.58%	SRR4852901

### DETs between the high and low temperature groups

**Figure 2:**
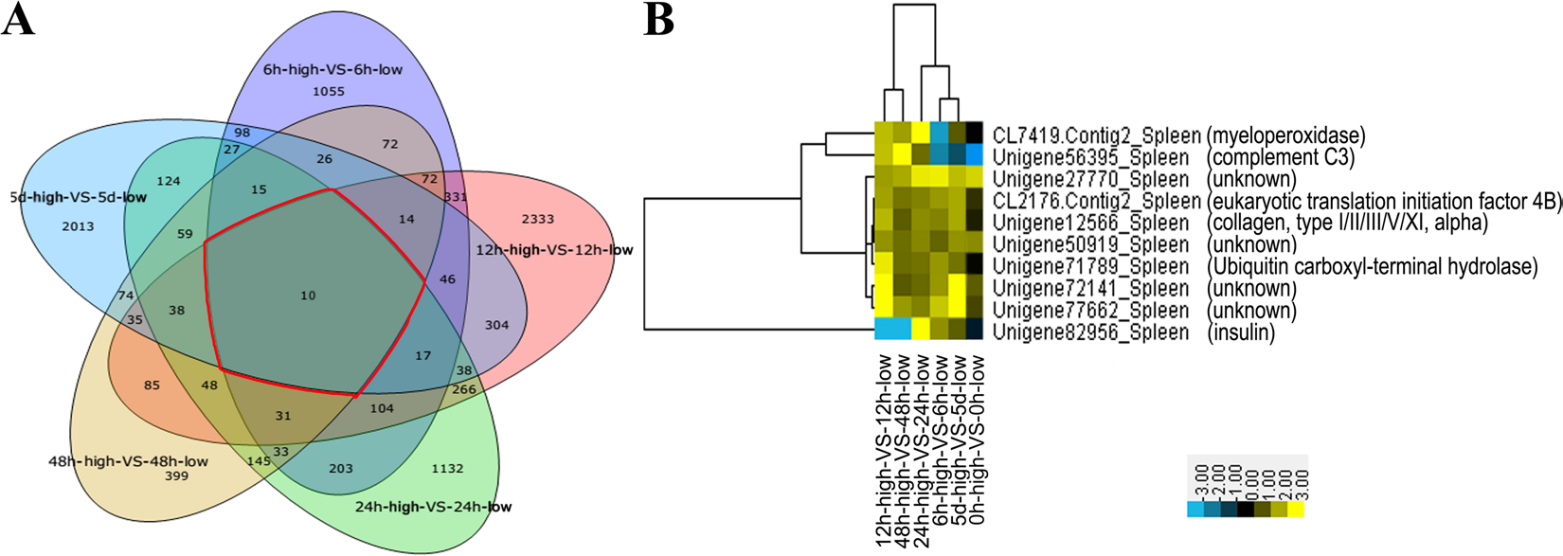
DETs comparison between the high and low temperature groups. (**A**) Venn charts for the number of comparisons of DETs. The region outlined in red represents the number of DETs shared by all five time-points after poly I:C-injection. (**B**) Hierarchical clustering of the 10 shared DETs. Yellow indicates up-regulated expression, meaning that the expression in the high temperature group was increased compared with that in the low temperature group. Blue indicates down-regulated expression. The corresponding number represents the value of log2 (fold_change).

The DETs between the high and low temperature groups at each time-point post poly I:C-injection were analyzed, providing a more integrated view of the impact of elevated temperature on the turbot facing pathogen attacks. There were 2,254, 3,772, 2,290, 1,156, and 2,938 DETs identified between the high and low temperature groups at 6 hpi, 12 hpi, 24 hpi, 48 hpi and 5 dpi, respectively. To capture the valid molecular response from these massive and variable expression data, we filtered these DETs by temporal comparison, and the Venn chart showed that 10 were shared by the DETs at all time-points (Fig. 2A). The annotations and expression patterns of the 10 DETs are illustrated in Fig. 2B. According to the molecular function, the 10 DETs are involved in immunity (myeloperoxidase, complement C3), metabolism (insulin), cytoskeleton (collagen), translation and protein degradation (eukaryotic translation initiation factor 4B, ubiquitin carboxyl-terminal hydrolase), and four unannotated DETs. In order to eliminate the genes only responding to the elevated temperature, DETs between the control group (0 h-high and 0 h-low) were analyzed. And three of the ten DETs shared by all time-points were also identified as DETs between 0 h-high and 0 h-low, including complement C3 and two unannotated DETs (Fig. 2B). This meant that other seven DETs were involved in the response changes to poly I:C under different temperature.

### General information of the proteome

The result of transcriptomic PCA (Fig. 1) indicated the importance of 24 hpi, thus, the 24 h-high and 24 h-low samples were applied to the proteomics analysis. In total, 6,295 unique peptides were identified from 223,110 spectra, corresponding to 2,436 proteins. The mass spectrometry proteomics data for the iTRAQ reagent labeling were deposited to the ProteomeXchange Consortium (http://proteomecentral.proteomexchange.org) via the PRIDE^34^ partner repository with the dataset identifier PXD005293.

### DEPs between the high and low temperature groups

The DEPs between the high and low temperature groups at 24 hpi were analyzed. There were 541 DEPs identified, among which 361 DEPs were up-regulated and 180 DEPs were down-regulated. According to the results of pathway enrichment analysis, these DEPs could be enriched to the pentose phosphate pathway, glutathione metabolism, type II diabetes mellitus, insulin signaling pathway, protein processing in the endoplasmic reticulum, leukocyte transendothelial migration, complement and coagulation cascades, natural killer cell mediated cytotoxicity, B-cell receptor signaling pathway, T-cell receptor signaling pathway, and the NF-kappa B signaling pathway.

### Correlation between DETs and DEPs

**Figure 3:**
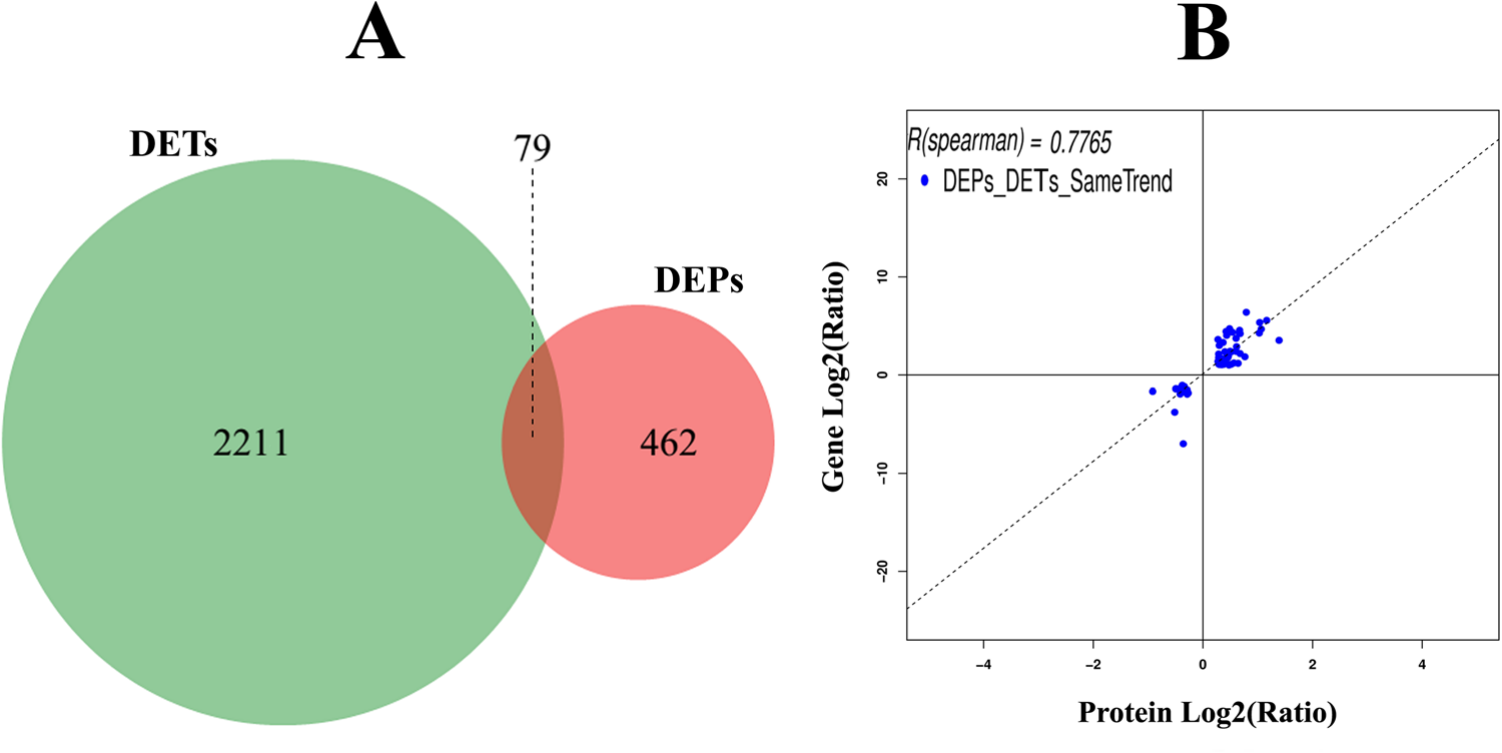
Correlation analyses between DEPs and DETs for 24 hpi. (**A**) Venn charts for the number of comparisons between DEPs and DETs. (**B**) Scatter plots of 71 “DEPs_DETs_SameTrend”, which had the same expression pattern at both the mRNA and protein levels (up- or down-regulated) among the 79 shared DEPs/DETs.

In total, 541 DEPs and 2,290 DETs were detected in the comparison of “24 h-high vs. 24 h-low”, among which 79 were shared (Fig. 3A). Among these 79 DETs/DEPs, 71 had the same expression trend at both the mRNA and protein levels, with a Spearman correlation coefficient of 0.7765 (Fig. 3B). Annotations of these 71 genes/proteins are listed in Table 2. According to the molecular function, these 71 correlated DEPs/DETs were mainly involved in immunity, metabolism, endoplasmic reticulum, cytoskeleton, digestion, and red blood cells. Compared with the 24 h-low, the expression levels of almost all correlated DEPs/DETs involved in immunity, metabolism, endoplasmic reticulum and cytoskeleton were up-regulated in the 24-high group, while the expression levels of all correlated DEPs/DETs involved in digestion and red blood cells were down-regulated in the 24-high group (Table 2). Among these 71 correlated DEPs/DETs, 21 were also identified as DETs between 0 h-high and 0 h-low, including two related to immunity, two related to metabolism, two related to cytoskeleton, six related to digestion, two related to red blood cells and seven related to other physiological function (Table 2). All the six digestion-related DEPs/DETs were also identified as DETs between 0 h-high and 0 h-low, which implied that the expression changes in digestion under different temperature at 24 hpi were likely just the response to the elevated temperature and not involved in the immune response to poly I:C.

**Table 2:** Correlated differentially expression transcripts/proteins for the comparison between 24 h-high and 24 h-low.

Correlation ID	Annotation	Protein fold-change	Gene fold-change	Expression pattern
	**Immunity**			
CL7419.Contig2_Spleen	myeloperoxidase	1.60	18.57	+
Unigene53474_Spleen	leukocyte elastase	1.58	20.13	+
CL5916.Contig1_Spleen	collagenase 3	2.05	40.76	+
Unigene62437_Spleen*	gelatinase	2.04	19.29	+(−)
Unigene41624_Spleen	macrophage mannose receptor 1	1.52	13.29	+
Unigene58435_Spleen	C-type lectin A	1.22	2.70	+
Unigene13731_Spleen	lipopolysaccharide-binding protein	1.43	5.12	+
Unigene12659_Spleen	glutathione peroxidase 4b	1.30	2.90	+
Unigene83002_Spleen*	leukocyte cell-derived chemotaxin-2	1.32	3.17	+(−)
	**Metabolism**			
	***insulin signaling pathway***			
Unigene82956_Spleen	insulin	1.21	12.25	+
CL8391.Contig3_Spleen	glycogen phosphorylase, liver form-like	1.59	23.41	+
Unigene71406_Spleen	starch phosphorylase	1.23	2.08	+
Unigene61349_Spleen	neuroendocrine convertase 2	1.34	21.54	+
	***pentose phosphate pathway***			
CL705.Contig1_Spleen	fructose-1,6-bisphosphatase 1	2.62	11.53	+
Unigene45470_Spleen	glucose-6-phosphate 1-dehydrogenase	1.60	4.52	+
Unigene71455_Spleen	phosphoglucomutase-1	1.41	5.32	+
Unigene12534_Spleen	6-phosphogluconate dehydrogenase	1.38	3.83	+
Unigene51900_Spleen	transketolase	1.33	3.28	+
Unigene51899_Spleen	transketolase-like protein 2-like isoform 1	1.25	2.97	+
Unigene54495_Spleen	hexokinase-2	1.70	3.60	+
CL8392.Contig3_Spleen	hexokinase-1	1.28	2.18	+
	***others***			
Unigene58275_Spleen	pyruvate kinase muscle isozyme-like isoform 1	1.31	2.94	+
Unigene52868_Spleen	leukotriene A-4 hydrolase-like	1.52	5.29	+
Unigene62971_Spleen	fatty acyl-CoA hydrolase precursor	1.53	7.37	+
Unigene42320_Spleen*	low choriolytic enzyme precursor	2.24	47.21	+(−)
Unigene1597_Spleen	isocitrate dehydrogenase [NADP] (cytoplasmic)	1.22	2.16	+
CL6409.Contig1_Spleen	phosphoglucose isomerase	1.39	2.00	+
Unigene69989_Spleen	6-phosphofructokinase, liver type	1.32	5.15	+
Unigene28736_Spleen	UDP-glucose 4-epimerase	1.27	2.08	+
CL7818.Contig2_Spleen	N-acylglucosamine 2-epimerase	1.25	2.41	+
CL1107.Contig2_Spleen*	betaine-homocysteine S-methyltransferase 1	0.78	0.36	−(−)
	**Cytoskeleton**			
CL729.Contig9_Spleen	myosin-9	2.09	25.51	+
Unigene57221_Spleen	myosin-9-like	1.40	26.23	+
Unigene73171_Spleen	spectrin beta chain, non-erythrocytic 1	1.23	3.61	+
Unigene55965_Spleen	annexin A2	1.25	2.81	+
Unigene56014_Spleen*	tropomyosin2	0.7	0.07	−(−)
Unigene55558_Spleen*	desmin	0.78	0.01	−(−)
	**Digestion**		1	
Unigene44879_Spleen*	carboxypeptidase A2 precursor	0.83	0.29	−(−)
Unigene40703_Spleen*	carboxypeptidase B	0.82	0.35	−(−)
Unigene40704_Spleen*	carboxypeptidase B	0.71	0.38	−(−)
CL1311.Contig3_Spleen*	trypsinogen	0.82	0.26	−(−)
CL8702.Contig1_Spleen*	astacin like metallo-protease	0.79	0.44	−(−)
Unigene66855_Spleen*	bile salt-activated lipase	0.76	0.42	−(−)
	**Red blood cell**			
Unigene67404_Spleen*	embryonic beta-type globin	0.77	0.44	−(−)
Unigene74254_Spleen	uroporphyrinogen-III synthase	0.77	0.48	−
Unigene67401_Spleen*	hemoglobin beta-2 subunit	0.77	0.40	−(−)
	**endoplasmic reticulum**			
Unigene67784_Spleen	endoplasmic reticulum oxidoreductin 1-like	1.22	2.32	+
Unigene26780_Spleen	thioredoxin domain-containing protein	1.42	2.10	+
Unigene58307_Spleen	methionine sulfoxidereductase	1.56	2.27	+
Unigene27533_Spleen	immediate early response 3-interacting protein 1	1.25	2.07	+
	**Others**			
Unigene68017_Spleen	inositol monophosphatase 1	1.73	83.75	+
Unigene65789_Spleen	carcinoembryonic antigen-related cell adhesion molecule 5	1.45	20.39	+
Unigene51827_Spleen	rho-related GTP-binding protein RhoG	1.26	2.59	+
Unigene65161_Spleen	coagulation factor VIII	1.23	8.04	+
Unigene82978_Spleen	sorcin	1.21	2.58	+
Unigene50212_Spleen*	glutathione S-transferase theta b	1.37	3.39	+(−)
Unigene46347_Spleen	phosphatidylethanolamine-binding protein 1	1.22	3.38	+
Unigene80941_Spleen	mitogen-activated protein kinase 6	1.43	2.12	+
Unigene81875_Spleen	sodium/potassium-transporting ATPase	1.30	2.10	+
Unigene67496_Spleen*	apolipoprotein Bb precursor	1.22	4.37	+(+)
Unigene30475_Spleen	Cysteine and glycine-rich protein 1	1.29	2.26	+
Unigene83050_Spleen	BTB/POZ domain-containing protein KCTD12	1.48	2.32	+
Unigene58708_Spleen	C6 or f115	1.39	2.07	+
CL5512.Contig2_Spleen	unnamed protein product	1.35	16.39	+
CL5626.Contig2_Spleen	unnamed protein product	1.29	9.90	+
CL7071.Contig1_Spleen	unnamed protein product	1.27	2.05	+
Unigene71460_Spleen*	DNA polymerase III polC-type	0.79	0.40	−(−)
CL5246.Contig1_Spleen*	C2 domain protein	0.79	0.32	−(−)
CL5246.Contig2_Spleen*	C2 domain protein	0.53	0.32	−(−)
CL4068.Contig2_Spleen*	cell adhesion molecule 4	0.75	0.27	−(−)
Unigene29996_Spleen*	unnamed protein product	0.79	0.43	−(−)

Note: “+” outside the brackets means that the expression in 24 h-high is up-regulated than that in 24 h-low, “−” outside the brackets means that the expression in 24 h-high is down-regulated than that in 24 h-low. “*” means that the gene is also a DET between 0 h-high and 0 h-low. “+” or “−” inside the brackets indicates that the expression in 0 h-high is up- or down-regulated than in 0 h-low.

Notably, myeloperoxidase (MPO) and insulin were DETs shared by all five time-points between high and low temperature (Fig. 2) and also had a consistent expression trend as DEPs at 24 hpi, implying the obvious influence of elevated temperature on the immunity and metabolism in the immunostimulated fish.

The results of correlation analysis (Table 2) showed that there were nine correlated DEPs/DETs involved in the function of immunity, including one chemotaxin (leukocyte cell-derived chemotaxin-2), three opsonins (macrophage mannose receptor 1, C-type lectin A, lipopolysaccharide-binding protein), three proteinases (leukocyte elastase, collagenase 3, gelatinase) and two peroxidases (MPO, glutathione peroxidase 4b). These immune-related genes were mainly involved in neutrophils. Leukocyte cell-derived chemotaxin-2 acts as a chemotactic factor of neutrophils^35^. Leukocyte elastase, gelatinase, collagenase and MPO are the components of neutrophils^36–40^. In addition, there were 22 correlated DEPs/DETs involved in the function of metabolism, among which four correlated DEPs/DETs participated in the “insulin signaling pathway”, and eight correlated DEPs/DETs participated in the “pentose phosphate pathway”. The information underlying these correlated DEPs/DETs will be elaborated in the discussion section.

### Oxidative damage

**Figure 4:**
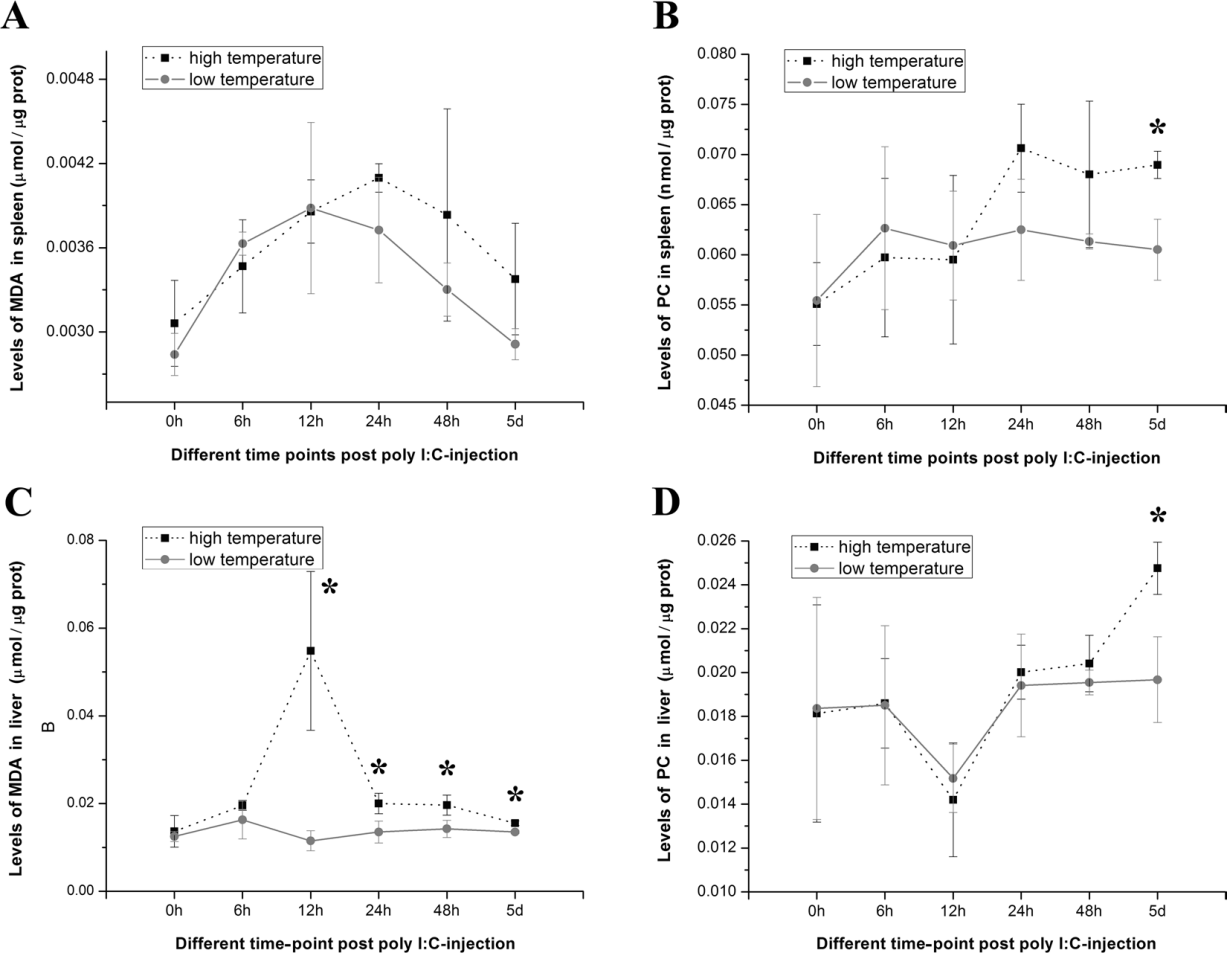
Comparisons of the MDA or PC content in the spleen and the liver of poly I:C-stimulated turbots between high and low temperature. The asterisk (*) indicates that there was a significant difference in the MDA or PC content between the high and low temperature group (*P* < 0.05).

Changes in the MDA content in the spleen of turbots after poly I:C-injection under high or low temperature are shown in Fig. 4A. The mean level of MDA under high temperature was higher than that under low temperature after 12 hpi, although the difference was not significant (*P* > 0.05). Changes in the PC content in the spleen of turbots after poly I:C-injection under high or low temperature are shown in Fig. 4B. The mean level of PC under high temperature increased after 12 hpi compared to that under low temperature, and the increase became significant at 5 dpi (*P* < 0.05). In order to understand the potential oxidative damage to other tissue than spleen, changes in the MDA and PC content after poly I:C-injection under high or low temperature were also detected in the liver of turbots, and the results were shown in Fig. 4C and D. The MDA content in the liver was significantly higher after 6 hpi under high temperature than low temperature (*P* < 0.05). And the PC content in the liver was significantly higher at 5 dpi under high temperature compared to low temperature (*P* < 0.05). In addition, the NADPH content in the spleen was also assayed and exhibited a significantly higher level at 24 hpi and 48 hpi under high temperature than low temperature (*P* < 0.05) (Supplementary Fig. S1).

## Discussion

Although a relationship has been implied between thermal stress and depressed disease resistance in fish^14,18,41^, comprehensive data on the molecular machinery that could finely explain this relationship are still limited. In this study, based on both transcriptomic and proteomic analyses, a number of molecular differentially expressed in the immunostimulated turbot between high and low temperature were identified, which would be helpful for showing the synergies between thermal stress and disease in the fish and predicting the negative impacts of elevated temperature on the turbot facing pathogen attacks.

### Acute response to poly I:C (before 24 hpi) implied the enhanced immunity, metabolism and oxidative stress by the elevated temperature

Poly I:C could induce an antiviral state in various species including fish^29,30^. The result of PCA showed that global transcriptional change between the high and low temperature groups was more drastic before 24 hpi than after 24 hpi (Fig. 1), suggesting that it was the acute response period before 24 hpi. To learn more about the acute response difference under different temperatures, we analyzed the differentially expressed genes at both the mRNA and protein levels between high and low temperature at 24 hpi. The results showed that the expression of 71 genes was consistently and significantly changed at both the mRNA and protein levels (Table 2), among which all immune-related genes exhibited up-regulated expression and were mainly involved in neutrophils. Neutrophils play important roles in initiating an inflammation response in the fish^42^. Neutrophils could also induce a respiratory burst response^43^. The increased expression of neutrophil-related genes suggested the enhanced inflammatory and respiratory burst response to poly I:C by elevated temperature. Some common genes related to the immune response to poly I:C, e.g. the genes encoding Mx proteins and beta2-microglobulin^30,44^, could be found in the DETs list of “24 h-low vs. 0 h-low”, while they did not show the differential expression between 24 h-high and 24 h-low, suggesting that the common immunity to poly I:C did not be influenced obviously by the elevated temperature.

Besides immunity, the expression of metabolism-related genes was also influenced, especially the genes involved in the “pentose phosphate pathway” (PPP) and “insulin signaling pathway” (Table 2). PPP is the major source of NADPH *in vivo*^45^, and NADPH is required for the efficient generation of reactive oxygen species (ROS) by neutrophils during the respiratory burst^35^. The increased expression of PPP-related genes by the elevated temperature was consistent with the significantly higher level of NADPH under high temperature than low temperature (Fig. S1), and further suggested the enhanced respiratory burst by neutrophils and the resultant oxidative stress on the fish. Insulin, as a powerful metabolic hormone, plays a central role in decreasing the plasma glucose level. The up-regulated expression of insulin under the high temperature implied an elevated plasma glucose level. In fish, the increase in plasma glucose has been used as an indicator of stress^46^. These results suggested that changes in the metabolism occurred in the poly I:C-stimulated turbot under the elevated temperature.

Endoplasmic reticulum stress (ER stress)-associated genes were also up-regulated in the high temperature group (Table 2). ER stress is a cellular stress response activated by the accumulation of unfolded or misfolded proteins in the endoplasmic reticulum^47^. ER stress and oxidative stress are intimately interrelated, and the generation of ROS could affect ER homeostasis and protein folding inside^48^. Among these differentially expressed ER stress-associated genes, methionine sulfoxidereductase and thioredoxin domain-containing protein have been reported to be related to the ER stress response against oxidative damage^49,50^. These results suggested that the elevated temperature aggravated the ER stress response in the poly I:C-stimulated turbot, which were consisted with the enhanced neutrophil-mediated respiratory burst and may be related to the oxidative stress.

Cytoskeleton- and red blood cell (RBC)-related genes also showed significantly different expression levels in the high temperature group compared with those in the low temperature group (Table 2). Oxidative stress affecting the cytoskeleton has been reported^51^, and oxidative stress could also lead to oxidative hemoglobin denaturation followed by RBC destruction^52^. Therefore, the significantly different expression levels of cytoskeleton- and RBC-related genes in the turbot may be also related to the oxidative stress.

DETs and DEPs between the high and low temperature group at 24 hpi suggested that high temperature enhanced fish immunity to poly I:C, especially neutrophil-mediated immunity and the resultant oxidative stress (e.g. the oxidative stress on protein, cytoskeleton and heme). Furthermore, it was suggested that high temperature disturbed the metabolism in the poly I:C-stimulated turbot, especially NADPH and glucose metabolism.

### Long period response to poly I:C (from 6 hpi to 5 dpi) implied a persistently enhanced MPO-mediated oxidative stress by the elevated temperature

Our results suggested that the neutrophil-mediated respiratory burst response in the poly I:C-injected turbots were enhanced by the elevated temperature at 24 hpi. Prolonged respiratory burst are stressful, which could damage turbot tissues and ultimately threaten survival. We further analyzed the global transcriptional expression changes between high and low temperature during a long period from 6 hpi to 5 dpi (i.e., 6 hpi, 12 hpi, 24 hpi, 48 hpi and 5 dpi), and the results showed that 10 DETs, including MPO, ubiquitin carboxyl-terminal hydrolase (UCH), collagen, insulin, complement C3, eukaryotic translation initiation factor 4B, and four unannotated DETs, were shared by all time points (Fig. 2). Except complement C3 and two unannotated DETs which were also DETs between 0 h-high and 0 h-low, other seven DETs were considered to be involved in the temperature-mediated response changes to poly I:C.

MPO is a peroxidase that is mainly expressed in neutrophils^39,40^ and plays an important role in the immunity of teleost fish^53^. It has been widely accepted about the correlation between MPO and oxidative damage. Toxic oxidants generated by the MPO-H_2_O_2_-halide system (e.g., hypochlorous acid and tyrosyl radical) are powerful and nonspecific, able to attack normal tissues and induce oxidative damage^39,54^. Except for MPO, UCH has also been considered as a biomarker for oxidative stress^55^. The significantly increased mRNA of MPO (from 12 hpi to 5 dpi) and UCH (from 6 hpi to 5 dpi) by the elevated temperature (Fig. 2B) revealed the sustained up-regulation of MPO and UCH in the immunostimulated turbots under the high temperature, which increased the odds of emergence of oxidative damage.

MPO-mediated oxidative injury has been reported to contribute to the disease of tissue fibrosis^39^. Collagen was reported to be dramatically induced under pathophysiological conditions of tissue fibrosis in mammals^56,57^. Our results showed that the transcriptional expression of collagen was significantly increased under high temperature compared to under low temperature (Fig. 2B), further implying the potential tissue injury by MPO in the poly I:C-stimulated turbot under high temperature.

Besides, a positive correlation between MPO, insulin resistance and metabolic disorder has been suggested^58^, thus, it is tempting to speculate that the significantly different expression of insulin from 6 hpi to 5 dpi between high and low temperature was related to insulin resistance induced by MPO-mediated oxidative stress. Alternative, the expression changes of insulin might also be related to the changes of energy consumption caused by the enhanced immunity in the infected turbot under high temperature.

Our results suggested that the elevated temperature aggravated the MPO-mediated oxidative stress in the poly I:C-stimulated turbot that lasted for at least 5 days, thus potentiating the oxidative damage. Lipids and proteins are significant targets for oxidative attack. To confirm the oxidative damage, we compared the level of MDA and PC in the spleen and the liver of poly I:C-stimulated turbots under different temperatures. The results showed that the PC content in the spleen and the liver under high temperature increased significantly at 5 dpi (*P* < 0.05) compared to that under low temperature (Fig. 4B,D). The changes of MDA contents were different between the spleen and the liver. In the spleen, there was no significant difference in the MDA level at each time-point post-injection between high temperature and low temperature (Fig. 4A). This result may be due to the significant increase in glutathione peroxidase 4 (Table 2), a peroxidase mainly utilizing lipid hydroperoxides as substrates^59^ that buffers the oxidative damage to lipids. In the liver, significant increase of MDA content under the high temperature were detected after 6 hpi (Fig. 4C), which might be related to the high lipid reserve in the liver and the resultant higher sensitiveness to the lipid oxidation. The oxidative damage data seemed highly variable, which may be the results of individual difference, tissue difference, the struggle to maintain homeostasis, and so on. Anyway, significant higher content of PC or MDA implied the greater oxidative damage in the infected turbot at higher temperature. Based on these results, it was suggested that sustained up-regulation of MPO by the elevated temperature increased the odds of emergence of oxidative damage in the immunostimulated turbots.

### Potential mechanisms linking thermal stress and depressed disease resistance

Based on the data of transcriptome and proteome, it was notable that MPO and insulin were DETs shared by all five time-points post poly I:C-injection between high and low temperature (Fig. 2) and also had a consistent expression trend as DEPs at 24 hpi, meanwhile for the control groups MPO and insulin were not DETs between 0 h-high and 0 h-low. Although we did not analyze the PBS-injected turbots from 6 hpi to 5 dpi, no report indicated that the expression of MPO or insulin would change significantly due to the injection of PBS. Furthermore, based on the data by Hori *et al*.^60^, the 290 DETs in the spleen of poly I:C-injected cod at 6 hpi between high and low temperature did not exhibit significantly differential expression in the PBS-injected cod at 6 hpi, the same to the 339 DETs at 24 hpi (shown by heat-map). The differential expression of MPO and insulin provided a clue about the potential mechanism linking thermal stress and depressed disease resistance.

Our results implied that for the turbot facing the pathogen attack, high temperature enhanced cell-mediated immunity, especially neutrophil-involved microbicity by MPO. The increase in MPO lasted for at least 5 days. Similar results were reported in rainbow trout, showing that after intraperitoneal immunization with *Aeromonas salmonicida*, the activation of the leucocyte populations of rainbow trout was temperature dependent. And the high temperature compared with the optimum temperature induced the stronger activation of granulocytes (mainly neutrophils), which lasted for at least 27 days^61^. Turbots may also experience a similar long period of neutrophil increase and a resultant MPO increase. MPO is close related with oxidative damage. Although MPO plays a critical role in host defense against invading pathogens, essentially, any oxidizable group on the host, e.g. sulfhydryl groups, heme groups and unsaturated fatty acids can also be oxidized. In fact, ER stress-, RBC- and cytoskeleton-associated genes showed significantly different expression levels in the immunostimulated turbots under different temperature, which were consistent with the aggravated oxidative stress. Significant increased oxidative damage in protein (i.e. PC) and lipid (i.e. MDA) were also induced by the elevated temperature in the immunostimulated turbots. Prolonged MPO-mediated oxidative stress may be extremely detrimental, thus threatening survival, which we thought was one potential mechanism inducing the thermo-linked epizootic. And more evidence on the relationship between oxidative stress and thermo-linked epizootic should be provided in the future, e.g. detection of other biomarker of oxidative damage, correlation analysis between oxidative damage and mortality.

The immune response and metabolic regulation are highly integrated, and the proper function of each is dependent on the other^62^. A positive correlation among MPO, insulin resistance and metabolic disorder has been suggested^58,63^. Interestingly, our results showed that besides MPO, insulin was another important differentially expressed gene. Whatever the reason, the expression changes of insulin implied that elevated temperature induced a prolonged instability state in metabolism, which would be detrimental to the turbot. Therefore, attentions should also be paid to the correlation between metabolic changes (e.g. metabolic disorder) and thermo-linked epizootic.

In the shadow of climate warming, this study will aid in showing the synergies between thermal stress and disease in the fish and comprehending the reason for the thermo-linked epizootic at the molecular level. Meanwhile, it will provide a reference for fish culture management when facing pathogen attacks. Further study is needed in the future to determine whether the mechanism discussed here applies to pathogens other than poly I:C.

## Materials and Methods

### Ethics statement

Experiments involving live animals were performed in accordance with the “Regulations for the Administration of Affairs Concerning Experimental Animals” promulgated by the State Science and Technology Commission of Shandong Province. The animal experiment was approved by the Science and Research Department of Institute of Oceanology, Chinese Academy of Sciences, and efforts were made to minimize the suffering of the animals.

### Fish and treatments

Turbot juveniles (50 ± 6 g) were collected from a turbot hatchery in Qingdao, China. The fish were divided into two temperature groups (~40 fish per group) and were separately placed in a 3000-L tank supplied with 15 °C or 21 °C aerated seawater (filtered, salinity ~30‰) and then were fed a commercial diet. After a 10-day acclimation, each fish was intraperitoneally injected with 0.5 mg of poly I:C (Sigma, USA). At each time-point post injection (i.e., 6 h, 12 h, 24 h, 48 h, and 5 d post-injection), for each temperature group (15 °C, 21 °C), spleens and livers were collected from six fish and were frozen immediately in liquid nitrogen until RNA or protein extraction. Thus, there were ten treatment groups, termed separately “6 h-high” (i.e., 6 h post poly I:C-injection [hpi] under 21 °C), “6 h-low” (i.e., 6 hpi under 15 °C), “12 h-high”, “12 h-low”, “24 h-high”, “24 h-low”, “48 h-high”, “48 h-low”, “5 d-high” and “5 d-low”. In addition, six fish were collected from each of the two temperature groups exactly before poly I:C-injection, and spleens and livers were dissected separately. These two groups were termed separately “0 h-high” and “0 h-low”, as the control groups. The fish were euthanized with tricaine methanesulfonate (Sigma, USA) before tissue collection to minimize suffering.

### Transcriptomic analyses (RNA-seq)

#### RNA extraction and quality control

Total RNA was extracted separately from the six spleen powder mixtures of each treatment using the SV Total RNA Isolation System (Promega, USA) according to the manufacturer’s instructions. RNA degradation and contamination was monitored on 1% agarose gels. RNA purity was checked using the NanoPhotometer spectrophotometer (Implen, GER). RNA integrity was assessed using the RNA Nano 6000 Assay Kit of the Bioanalyzer 2100 system (Agilent Technologies, USA) and was expressed as the RNA Integrity Number (RIN). Three micrograms of qualified RNA (RIN > 8) was used. RNA of the 10 treatments was applied to a library preparation for single-end RNA sequencing separately, and pooled RNA was applied to a library preparation for *de novo* transcriptome sequencing (paired-end RNA sequencing).

#### Library preparation and sequencing

The library was generated using the Illumina TruSeq™ RNA Sample Preparation Kit (Illumina, USA) following the manufacturer’s recommendations. The details are described in Yue *et al*.^64^. Next, the libraries were sequenced using an Illumina Hiseq. 2000 platform. All raw tag data were filtered, by which clean data were obtained by removing reads containing adapters, reads containing poly-N, and low-quality reads from raw data. All of the downstream analyses were based on clean data with high quality.

#### De novo transcriptome assembly and gene function annotation

Transcriptome assembly was accomplished using Trinity^65^, by which unigenes were obtained. Genes were annotated based on the following databases with a cutoff *E* value of 1.0 × 10^−5^: Nr (NCBI non-redundant protein sequences); Nt (NCBI non-redundant nucleotide sequences); COG (Clusters of Orthologous Groups of proteins); Swiss-Prot (A manually annotated and reviewed protein sequence database); KO (KEGG Orthology database); and GO (Gene Ontology).

#### Sequence mapping and gene expression quantification

The assembled *de novo* transcriptome was used as the reference database, and gene expression levels were estimated for each library. Briefly, the clean data of RNA sequencing were mapped back to the reference transcriptome by Bowtie v0.12.9, and the read count for each gene was obtained from the mapping results by RSEM^66^. Next, the RPKM (Reads Per Kilobase of exon model per Million mapped reads) of each gene, a typical parameter for estimating gene expression levels, was calculated based on the length of the gene and read count mapped to the gene^67^.

#### Differential expression analysis

For each sequenced library, the read counts were adjusted by the edgeR program package. Differential expression analysis between two treatments was performed using the DEGSeq R package (1.12.0) to detect the differentially expressed transcripts (DETs). *P* values were adjusted using the Benjamini-Hochberg procedure^37^. A corrected *P* value of 0.005 and log2 (fold_change) of 1 were set as the threshold for significantly differential expression, which was adopted widely in the *de novo* transcriptome. Hierarchical cluster analysis of DETs intersection was performed to assess the transcriptional pattern variations among different time points using Cluster 3.0^68^. Venn charts were drawn using the Venn Diagram R package to exhibit shared or specific differentially expressed genes between different pairwise comparisons. Principal component analysis (PCA) was performed using the Simca-P 8.0 software package (Umetrics, Sweden).

### Proteomic analyses (iTRAQ)

#### Protein extraction and quantification

For each treatment of “24 h-high” and “24 h-low”, total protein was extracted separately from two of the six spleens sampled (i.e., three biological replicates for each treatment). Briefly, spleens were ground in liquid nitrogen and were suspended in 10 volumes of lysis buffer containing 7 M Urea, 2 M thiourea, 0.1% CHAPS, and one piece/50 ml Protease Inhibitor Cocktail (Roche, Switzerland) and then were sonicated for 15 min and centrifuged at 15,000 g at 4 °C for 20 min. The protein concentration was determined by 2-D Quant Kit (GE Healthcare, UK).

#### Protein digestion and iTRAQ labeling

For each sample, 100 μg of protein was used and applied to the following process. The disulfide bond of the supernatant was reduced with 25 mM DTT at 60 °C for 1 h, cysteine was blocked with 55 mM iodacetamide in the dark for 45 min, and then the mixture was added to Millipore Amicon Ultra-15 (MWCO 10 k), followed by washing three times using iTRAQ Dissolution Buffer (AB Sciex, USA) and the addition of 4 μg of Trypsin Gold (Promega, USA) for protein digestion overnight at 37 °C. The resulting peptide solution was collected after centrifugation at 12,000 g for 20 min. The peptide solution was then labeled using the iTRAQ Reagent-8plex Multiplex Kit (AB Sciex, USA) according to the manufacturer’s instructions. Three biological replicates for two treatments were applied to the iTRAQ reagent (6-plex) labeling as follows: 24 h-low-1 (115), 24 h-low-2 (116), 24 h-low-3 (117), 24 h-high-1(118), 24 h-high-2 (119), and 24 h-high-3 (121). MALDI-TOF-TOF (AB Sciex 4800 Plus) was applied to check the labeling efficiency and quantitative accuracy.

#### Peptide fractionation and LC-MS/MS

The iTRAQ-labeled peptides were fractionated using the high-pH reversed-phase (RP) chromatography approach^69^, which was manipulated in an HPLC system (RIGOL L-3000). The fractioned peptides were collected and combined into 20 final fractions, which were applied to LC-MS/MS. In brief, the prepared sample was loaded on an EASY-nLC 1000 nano HPLC (Thermo Scientific, USA). The peptides were eluted onto an analytical C18 column (10 cm × 75 μm). The peptides were subjected to electrospray ionization followed by tandem mass spectrometry (MS/MS) in a Q-Exactive (Thermo scientific, USA). Intact peptides were detected in the Orbitrap. The electrospray voltage applied was 2.1 kV. Automatic gain control (AGC) was used to optimize the spectra generated by the orbitrap. For MS scans, the m/z scan range was 350–2000.

#### Database search, protein quantification and differential expression analysis

All of the LC-MS/MS raw data were converted to the Mascot generic Format (.mgf) by the thermo scientific tool Proteome Discoverer 1.3. Mascot version 2.4.1 (Matrix Sciences, UK)^70^ was used to search against the predicted protein database translated from the *S. maximus* transcriptome obtained in this study for peptide sequence assignments. To reduce the probability of false peptide identification, only peptides at a 95% confidence interval (*P* < 0.05) with a false discovery rate (FDR) estimation ≤1.04% were counted as being successfully identified. Each identified positive protein contained at least one unique peptide. IQuant software^71^ was used to quantitatively analyze the labeled peptides with isobaric tags. It integrates Mascot Percolator and advanced statistical algorithms to process the MS/MS signals generated from the peptides labeled by isobaric tags. Any protein changed with a 1.2-fold difference and a corrected *P* value < 0.05 would be designated as the differentially expressed protein (DEP). DEPs were then applied to a pathway enrichment analysis based on the KEGG database using KOBAS software.

### Correlation analyses between transcriptome and proteome

To comprehensively understand the molecular response of the immunostimulated turbot to the elevated temperature, transcriptome-proteome correlation analysis was performed. For the comparison between 24 h-high and 24 h-low, the genes showing significantly different expression at both the mRNA and protein levels were identified by screening the shared DETs and DEPs. These genes were divided into two groups, those with the same or the opposite expression trend, and each group was then subjected to Spearman correlation analysis. Venn charts were drawn to exhibit the relationship between DETs and DEPs.

### Detection of oxidative damage related parameters

Malondialdehyde (MDA) contents, a marker of oxidative damage to lipids^72^, and protein carbonyls (PC) contents, a marker of oxidative damage to proteins^73^, were measured in the spleen and the liver, respectively. For each of the ten treatment groups and two control groups, total protein was extracted separately from each of the six spleens or six livers. In brief, tissue combined with 9 times the volume of 0.01 M PBS (pH 7.2) was ground thoroughly on ice. The homogenate was centrifuged at 4 °C, and then the supernatant was collected as the tissue extract sample, followed by application to the following assays. The total protein concentration of each sample was determined using the 2-D Quant Kit (GE Healthcare, UK).

MDA concentrations were measured using a commercial kit purchased from Beyotime Institute of Biotechnology (China) based on the method described by Ohkawa *et al*.^74^. Briefly, 0.1 ml of each sample combined with 0.2 ml of MDA detection agent containing thiobarbituric acid (TBA) was heated in a boiling water bath for 15 min. After cooling to room temperature, the reaction mixture was centrifuged at 1000 g for 10 min. The absorbance of the supernatant (200 μl) at 532 nm was measured in a 96-well plate with an Infinite M1000 Pro spectrophotometer (Tecan, Switzerland). The levels of MDA were calculated from the standard curve based on known concentrations of MDA and were expressed as μmol/μg prot.

The PC concentration was measured using a PC ELISA kit (QIYBIO, China). Briefly, 50 μl of each sample was added to the well coated with PC antibody, and then 100 μl of HRP-conjugated PC antibody was added to each well. After a one-hour incubation at 37 °C and washing five times, 50 μl of chromogen solution A and 50 μl of chromogen solution B were added to each well. The absorbance of each well at 450 nm was measured in a 96-well plate using the Infinite M1000 Pro spectrophotometer (Tecan, Switzerland). The levels of PC were calculated from the standard curve based on known concentrations of PC and were expressed as nmol/μg prot.

Besides, the NADPH concentration, an oxidative stress-related parameter that correlated with pentose phosphate pathway, was also measured in spleens of the ten treatment groups and two control groups using a commercial kit purchased from Comin Biotechnology (China) based on the method described by Gibon and Larher^75^. The levels of PC of each sample were calculated based on the absorbance at 570 nm and were expressed as nmol/mg prot.

### Data accessibility

Clean data files of transcriptome have been deposited in SRA of NCBI, and can be found according to the SRA accession numbers listed in Table 1. The MS-based proteomic data have been deposited to ProteomeXchange Consortium and are available with identifier PXD005293.

## Electronic supplementary material

Figure S1
