# Supplementary material for: Integrated transcriptomic and proteomic analyses reveal potential mechanisms linking thermal stress and depressed disease resistance in the turbot Scophthalmus maximus
Source: Sci Rep. 2018 Jan 30;8:1896. doi: 10.1038/s41598-018-20065-1 (PMC5790011; doi:10.1038/s41598-018-20065-1)
Supplement: Supplementary file 1 — Figure S1 [file 41598_2018_20065_MOESM1_ESM.pdf]

Supplementary Information

Integrated transcriptomic and proteomic analyses reveal potential mechanisms linking thermal stress and depressed disease resistance in the turbot *Scophthalmus maximus*

Xin Yue[1], Pin Huan[1], Yonghua Hu[1], Baozhong Liu[1,2*]

[1] *Key Laboratory of Experimental Marine Biology, Institute of Oceanology, Chinese Academy of Sciences, 7 Nanhai Road, Qingdao 266071, China*

[2] *Laboratory for Marine Biology and Biotechnology, Qingdao National Laboratory for Marine Science and Technology, 1 Wenhai Road, Qingdao266000, China*

Fig. S1

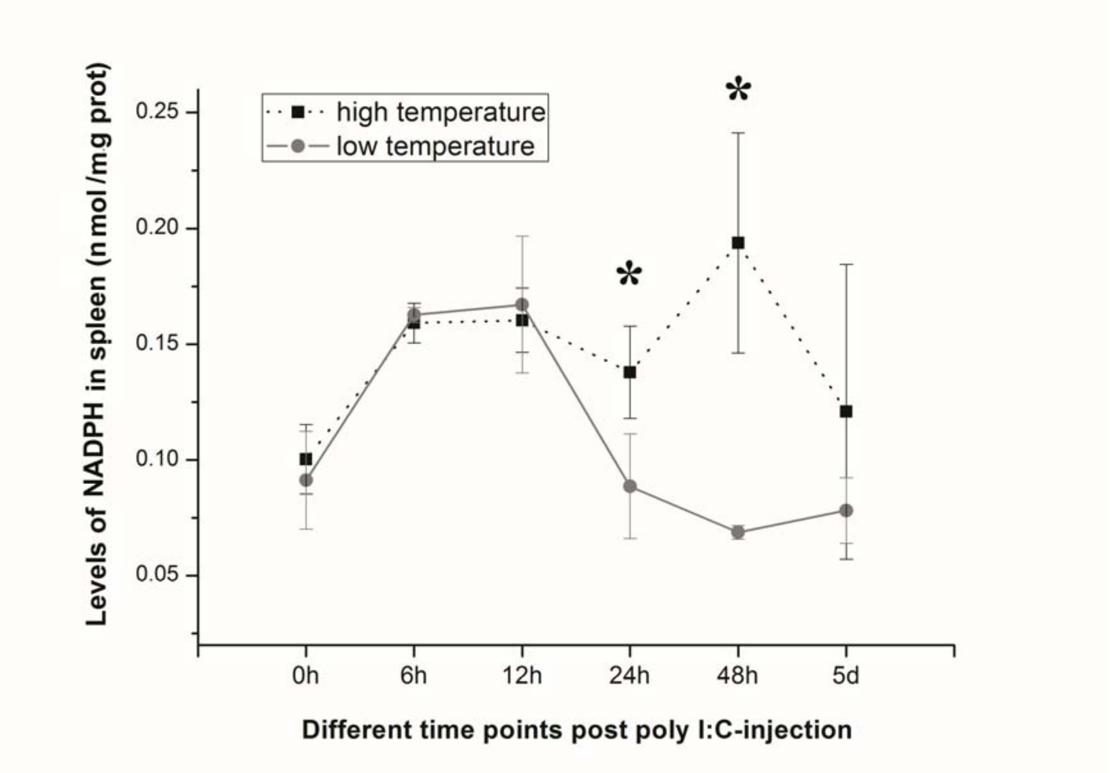

Fig. S1. Comparisons of the NADPH content in the spleen of poly I:C-stimulated turbots between high and low temperature. The asterisk (*) indicates that there was a significant difference in the NADPH content between the high and low temperature group (P<0.05).
